# An Epiphrenic Diverticulum with Previous Perforation Excised Laparoscopically

**DOI:** 10.1155/2024/3556567

**Published:** 2024-06-12

**Authors:** Amitabh Yadav, Samiran Nundy

**Affiliations:** Department of Surgical Gastroenterology and Liver Transplantation Sir Ganga Ram Hospital, New Delhi, India

## Abstract

An epiphrenic diverticulum (ED) is a rare pseudodiverticulum commonly located at the lower end of the oesophagus. Surgical treatment is only required in patients with worsening dysphagia or repeated chest infection due to microaspirations, and most patients are now treated with minimally invasive surgery (MIS) using the laparoscopic or thoracoscopic approach. Laparoscopic surgery is considered difficult in the presence of previous perforation of the diverticula owing to the intraperitoneal and mediastinal adhesions and is associated with an increased incidence of complications. We were able to perform a laparoscopic transhiatal resection safely on a patient who had a large ED with a wide neck and dense abdominal and mediastinal adhesions due to previous localized perforation.

## 1. Introduction

An epiphrenic diverticulum (ED) is a rare mucosal outpouching that can occur anywhere in the oesophagus but is commonly located in its distal part, 4–8 cm above the oesophagogastric junction [1]. It is a type of pseudodiverticulum that develops due to herniation of the mucosa and submucosa through a weak area in the muscularis layer of the oesophagus [2]. It is commonly detected on routine endoscopic or radiological evaluation, and up to 80% of patients are either asymptomatic or have very minimal symptoms. It accounts for less than 10% of all oesophageal diverticula, and the majority are treated conservatively [3]. For surgical resection, the laparoscopic transhiatal or thoracoscopic routes are now preferred over the conventional open thoracotomy approach. Laparoscopic surgery is practiced more than the thoracoscopic approach because of its wider availability and the larger number of trained specialists in this field. Previous articles have quoted that the laparoscopic approach was used for surgical resection of ED in 84% of patients, thoracoscopy in 14%, and a combined approach in 2% [4]. The location is also important for choosing an approach to treatment, as a diverticulum situated more than 10 cm away from the hiatus and with a wide neck usually requires a transthoracic route to dissect it from the mediastinal structures [5]. Dissection of the upper portion of the neck of the diverticulum is also difficult via the laparoscopic approach, especially if it is large and has pleural or mediastinal adhesions, and thoracoscopic assistance may be required to dissect it from the mediastinal structures [6]. The indications for thoracoscopy in ED include cases with mediastinal adhesions, large size, and a previously failed laparoscopic approach [4]. A postoperative leak is a serious complication and may be related to the large size of the diverticulum and difficult closure owing to the small operative space available via a thoracoscopy. The chances of a leak are greater after laparoscopy, especially if the size of the diverticulum is more than 9 centimeters, and thoracoscopic assistance may be required during resection in such cases [7]. We have successfully done a laparoscopic transhiatal diverticulectomy, myotomy, and a partial fundoplication on a large wide-necked ED with mediastinal and dense intra-abdominal adhesions due to a previous localized rupture.

## 2. Case Presentation

A 52-year-old man smoker without any comorbidities presented to us with worsening dysphagia to solids, regurgitation of previously taken food, and frequent respiratory infections for four months. He had an episode of severe chest and abdominal pain 8 months ago, which was diagnosed as oesophageal diverticulum with localized rupture on a contrast-enhanced CT scan. Since his chest condition was not optimal for surgery, he was treated conservatively. He was managed at an outside hospital and later referred to our institute which is a tertiary care teaching hospital.

## 3. Investigations

**Figure 1:**
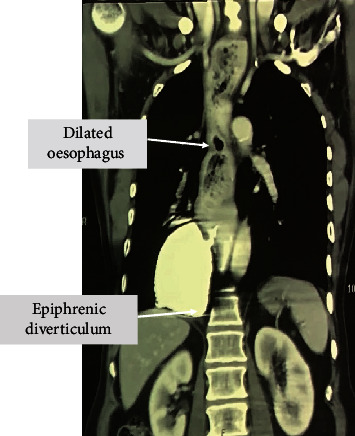
Preoperative CT scan showing oesophageal diverticulum and dilated oesophagus.

He was evaluated with an oral contrast swallow, an upper gastrointestinal (UGI) endoscopy, and a manometry. UGI endoscopy showed a dilated oesophagus throughout its entire length, with a large diverticulum at its lower end. Radiological evaluation with a contrast swallow showed absent contraction and a large diverticulum at the lower end of the oesophagus. Further, CECT chest and abdomen were suggestive of a markedly dilated oesophagus along its entire length with an ED (8.5 × 6.5 × 7.9) from D8 to D11 level on the right side of the oesophagus, with normal passage of contrast distal to it (Figure 1). Diffuse emphysematous change with no area of cavitation or consolidation was seen in the pulmonary parenchyma, mainly on the right side. Oesophageal manometry revealed a normal gastroesophageal (GE) junction morphology with increased basal pressure and incomplete relaxation of the lower oesophageal sphincter.

## 4. Treatment

**Figure 2:**
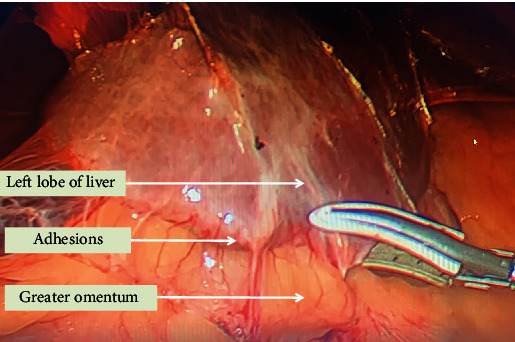
Intraoperative picture showing adhesions between left lobe of liver and greater omentum.

**Figure 3:**
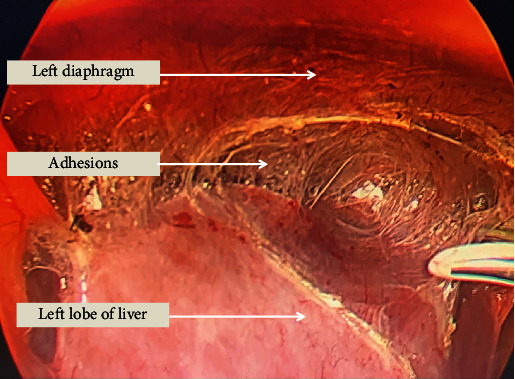
Intraoperative picture showing adhesions between left lobe of liver and left diaphragm.

**Figure 4:**
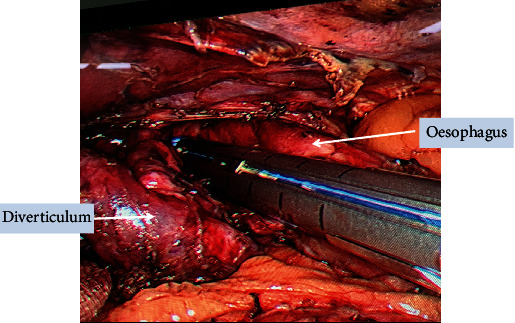
Intraoperative picture showing stapler used parallel to the oesophagus.

**Figure 5:**
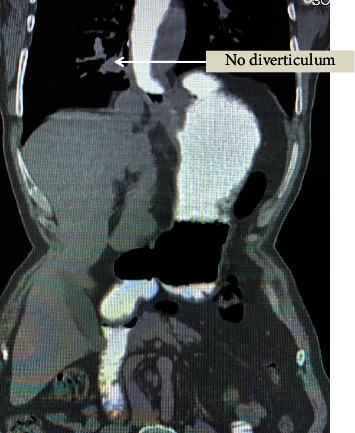
Immediate postoperative CT showing no outpouching or leak from oesophagus.

The patient was first optimized for his chest condition and then planned for surgery after proper informed consent. Since it was a large diverticulum with a wide neck, the preparation for thoracoscopy was also kept ready in the operating room. He was placed in the reversed Trendelenburg (30 degree) position with his legs split. A pneumoperitoneum was created, and a 10 mm supraumbilical camera port and two working ports, 10 mm on the right and 5 mm on the left side of the upper abdomen, were placed. The other 10 mm port was placed in the right hypochondrium to retract the left lobe of the liver. On entering the abdominal cavity, dense upper abdominal intraperitoneal adhesions between the diverticula, diaphragm, and the right pleural cavity were encountered, which probably were due to the previous sealed perforation (Figures 2 and 3). Meticulous adhesiolysis with blunt and sharp dissection, using a suction catheter and harmonic scalpel, was done. The oesophageal hiatus was dissected, both vagi were identified, and the lower end of the oesophagus was mobilized from the surrounding structures and looped. Because of the size of the diverticulum, the main problem in dissection was encountered while separating its superior pole from the mediastinal structures. After dissecting it from the mediastinal structures, a bougie was inserted orally into the oesophagus to safeguard the oesophageal lumen while firing a stapler. Three cartridges of 60 mm of laparoscopic staplers were fired, keeping them parallel and along the wide neck of the ED (Figure 4). No suture repair was done over the stapled line. The oesophageal lumen was seen from inside by UGI endoscopy, and a simultaneous leak test was also done with minimal insufflation. A cardiomyotomy was performed on the opposite side of the diverticulum (on the left side of the oesophagus, starting just distal to it) for 5 cm on the oesophageal side and 2 cm on the stomach. The hiatus was closed with a partial Dor fundoplication. A soft tube drain was placed near the oesophageal hiatus, and the patient was electively ventilated overnight in view of his preexisting chest condition. The blood loss was about 100 ml, and the duration of surgery was 290 minutes. He was extubated but kept in the postoperative intensive care unit for 2 days due to his previous chest condition. He was kept on sips of clear liquids from the next day, but the nasogastric tube was kept for 3 days. An oral contrast CT scan was done on the fourth postoperative day, which showed no leak, and he was started on a liquid diet (Figure 5). He was discharged on the 6th postoperative day on soft oral diet. No parenteral nutrition was given to him during hospital stay. Histopathology showed benign disease with no evidence of malignancy.

## 5. Outcome and Follow-Up

A check endoscopy was done one month later, which showed the absence of the diverticulum and no obstruction at the lower end of the oesophagus. He experienced reflux in the initial postoperative period, for which he was given proton pump inhibitors and prokinetics. He has been in the follow-up for last 22 months, and his symptoms prior to the surgery have been completely resolved.

## 6. Discussion

The incidence of an ED is about 1 : 500,000 per year in Western countries [3, 7]. It is more common in men, with a peak age of presentation between the sixth and seventh decades of life [8]. The diverticulum has an associated malignancy in about 0.6% of the cases, and the incidence of malignancy is greater in old age, in men, in those with a long history of disease, and in diverticula of more than 5 centimeters in size. Its most common location is on the right side of the lower oesophagus, which is found in about 70% of the patients. It is single in most of the patients, but in 15%, there may be multiple diverticula [9]. The cause behind its development is usually a raised intraluminal pressure due to a functional obstruction in the distal oesophagus, and the common causes are achalasia, a hypertensive distal oesophageal sphincter, and diffuse motility spasm [10]. Previous reports suggested that it is caused by oesophageal spasm in 38%, achalasia in 16%, and a nutcracker oesophagus in 8% of patients, and 27% of patients had normal oesophageal motility [11]. It can also be a postoperative complication of a tight fundoplication causing distal mechanical obstruction and a raised intraluminal pressure [12].

The literature has reported that 50-80% of patients have very minimal or no symptoms [4]. There is no correlation between the size of the diverticula and the symptoms they cause [11], but diverticula larger than 5 cm are usually symptomatic [13]. The symptoms are due to the underlying motility disorder rather than the diverticula itself, and they may vary from asymptomatic “detected incidentally” to worsening dysphagia, vomiting, and regurgitation. The other common presentations include aspiration pneumonitis due to frequent microaspirations. Symptoms like weight loss and melaena/hematemesis are considered worrisome and may indicate a malignant change. The other presentations may be due to compression of the surrounding structures [14]. The chances of developing complications during a lifetime in asymptomatic patients with ED are less than 10% [2, 14]. The complications, though rare, may arise due to its size, inflammation, or malignant change. These include microaspirations leading to aspiration pneumonitis, ulcerations, rupture, bleeding, fistulization with the surrounding organs, or malignant change. The present patient had fibrous adhesions between the left lobe of the liver, diaphragm, and pleural cavity on the right side. A contrast study helps in finding the location and size of the diverticulum, motility, and an associated stricture or lesion. Endoscopy usually confirms these findings and has 91% sensitivity when used alone [15]. Manometry is essential to know about the motor functions of the oesophagus. In patients with symptomatic reflux, pH monitoring is required to differentiate between true reflux and reflux from a diverticulum [16]. Some studies do not consider manometry and pH studies essential, as they believe that the distal motility disorder always exist [17].

Asymptomatic patients with normal manometry are managed conservatively. Nonsurgical treatment options include pneumatic dilatation or local botulinum toxin injections [18]. These are good alternatives to surgical treatment if the patient is unfit or unwilling to undergo surgery. The mere presence of a diverticulum and its size are not indications for surgical intervention. Surgery is recommended if the symptoms are severe like dysphagia, retention of food inside the diverticulum with vomiting or regurgitation, or aspiration pneumonitis [3]. A life-threatening complication of ED is aspiration pneumonitis, and some authors have suggested its excision in all cases owing to the risk of this complication. The proportion of patients requiring surgical intervention may vary from 0 to 40% [11]. Current opinion however, suggests that the surgery should be reserved for patients with severe symptoms as the risk of surgery outweighs the risk of aspiration pneumonitis [5]. The disease is so rare that only a few studies have been published on the available surgical options. Various surgical approaches have been proposed, depending on the surgeon's preference and expertise [19], and it can be done using an open or minimally invasive approach. In the past, the standard of surgical care for ED had been a transthoracic approach through a left thoracotomy [15], but the disadvantages of this approach included the morbidity due to the incision, the placement of a double lumen endotracheal tube, the lateral decubitus position, and a thoracic drain in the postoperative period [20]. MIS, including the laparoscopic transhiatal and thoracoscopic approaches, were introduced in the late nineties and are less morbid, associated with less postoperative pain, shorter hospital stay, and avoidance of the transthoracic drains [2].

The ED situated in the lower chest can be approached either from the chest or abdomen, but large neck diverticula reaching above the pulmonary vein are best dealt with a combined approach of thoracotomy/thoracoscopy for excision and a laparoscopic approach for myotomy and fundoplication [11]. Laparoscopy has better vision than thoracoscopy, and it has the advantages of easy access to the upper abdominal structures, a better visualized myotomy, the addition of fundoplication, and no need for single lung ventilation [19]. It is described as the best approach to perform all the three components of the surgery when the diverticulum is closer to the oesophageal hiatus and is less than 4 cm across the base [3, 15]. A recent article suggested that laparoscopic approach has been shown to have lower conversion rates to open surgery, improved dysphagia, shorter stay, and less reflux [21].

The goals of surgery include excision of the diverticulum, correction of the underlying motility disorder (oesophagogastric myotomy), and a fundoplication to prevent postoperative reflux. Diverticulectomy alone as a surgical procedure was performed in 100% of the patients, myotomy in 83%, and fundoplication in 85% of the patients in a previously published article [4]. Diverticulectomy with myotomy relieves symptoms in about 90% of patients [2]. The myotomy should be extended to the distal most area below the diverticulum, which is responsible for the distal obstruction. Myotomy resolves the distal obstruction in the oesophagus, reduces pressure and the chances of a subsequent leak, and reduces recurrence rates [5, 7]. Patients treated without myotomy had recurrence and leak rates of up to 20-24%. Symptomatic small diverticula can be managed by myotomy alone, as they shrink after the procedure [19]. Larger diverticula require intervention because of the risk of complications they carry. Combining a Dor fundoplication reduces the rate of postprocedure reflux from 48 to 9.5% [22]. Postoperative complications include leaks from the suture line or missed mucosal injuries during the myotomy, dysphagia due to an incomplete myotomy or tight wrap, and reflux. The commonest complication is a leak from the suture line, reported in 5-37% of patients [23]. The main risk factors for the leak are the use of two or more staple cartridges, a wide neck of the diverticulum, or its location in the mediastinum [11].

The reported morbidity rates after surgery are 5.3-50%, mortality up to 9%, and a leak rate of 16.6%. A successful outcome of the surgery is reported in 70% of cases, but the symptoms remain unchanged in 21% and worsen in 8% of cases in previous studies [4, 9]. The risk of postoperative acid reflux is seen in up to 60% of patients, which can be reduced by adding a partial fundoplication [9]. The outcome of the present patient is successful at 22-months of follow-up.

## 7. Learning Points

- Laparoscopic transhiatal dissection provides good exposure and can be safely performed even in a large ED with wide neck and a previous localized perforation
- The stapler line is better oriented in laparoscopy
- As the operative experience growing and the quality of the staplers improving, even a large diverticulum can be treated laparoscopically with a satisfactory outcome

## Data Availability

Data is available upon the corresponding author's request.
